# Exercise-Related Transient Abdominal Pain (ETAP)

**DOI:** 10.1007/s40279-014-0245-z

**Published:** 2014-09-03

**Authors:** Darren Morton, Robin Callister

**Affiliations:** 1 Faculty of Education and Science, Lifestyle Research Centre, Avondale College of Higher Education, PO Box 19, Cooranbong, NSW 2265 Australia; 2 Faculty of Health and Medicine, and Priority Research Centre in Physical Activity and Nutrition, School of Biomedical Sciences and Pharmacy, University of Newcastle, Callaghan, NSW 2308 Australia

## Abstract

Exercise-related transient abdominal pain (ETAP), commonly referred to as ‘stitch’, is an ailment well known in many sporting activities. It is especially prevalent in activities that involve repetitive torso movement with the torso in an extended position, such as running and horse riding. Approximately 70 % of runners report experiencing the pain in the past year and in a single running event approximately one in five participants can be expected to suffer the condition. ETAP is a localized pain that is most common in the lateral aspects of the mid abdomen along the costal border, although it may occur in any region of the abdomen. It may also be related to shoulder tip pain, which is the referred site from tissue innervated by the phrenic nerve. ETAP tends to be sharp or stabbing when severe, and cramping, aching, or pulling when less intense. The condition is exacerbated by the postprandial state, with hypertonic beverages being particularly provocative. ETAP is most common in the young but is unrelated to sex or body type. Well trained athletes are not immune from the condition, although they may experience it less frequently. Several theories have been presented to explain the mechanism responsible for the pain, including ischemia of the diaphragm; stress on the supportive visceral ligaments that attach the abdominal organs to the diaphragm; gastrointestinal ischemia or distension; cramping of the abdominal musculature; ischemic pain resulting from compression of the celiac artery by the median arcuate ligament; aggravation of the spinal nerves; and irritation of the parietal peritoneum. Of these theories, irritation of the parietal peritoneum best explains the features of ETAP; however, further investigations are required. Strategies for managing the pain are largely anecdotal, especially given that its etiology remains to be fully elucidated. Commonly purported prevention strategies include avoiding large volumes of food and beverages for at least 2 hours prior to exercise, especially hypertonic compounds; improving posture, especially in the thoracic region; and supporting the abdominal organs by improving core strength or wearing a supportive broad belt. Techniques for gaining relief from the pain during an episode are equivocal. This article presents a contemporary understanding of ETAP, which historically has received little research attention but over the past 15 years has been more carefully studied.

## Key Points

**Table:** 

ETAP is a well known and common condition that, until a spate of investigations over the past 15 years, has historically received limited research attention.
The characteristics of ETAP are reasonably well understood but the mechanism responsible for the pain remains to be fully elucidated.
Further studies are required to determine the etiology of ETAP so as to inform effective strategies for managing the condition.

## Introduction

Exercise-related transient abdominal pain (ETAP) [1], colloquially referred to as ‘stitch’ [2] and ‘side ache’ [3, 4], is a common condition observed in many sporting activities [1]. References to the pain can even be found in the works of Shakespeare and Pliny the Elder [3, 5]. Despite its widespread occurrence, ETAP has historically received little research attention and hence has been poorly understood. As shown in Table 1, which cites the key studies of ETAP that have produced novel findings, several studies of the pain appeared in the medical literature in the first half of the 20th century [2, 3, 5–9], but the condition was subsequently not investigated for almost 50 years. Over the past 15 years a renewed interest in ETAP has resulted in a series of publications that have clarified many aspects of the ailment. This review presents a contemporary understanding of the prevalence and characteristics of ETAP, factors related to its provocation, potential mechanisms responsible for its manifestation, and strategies for preventing and managing the pain.

**Table 1:** Key studies of exercise-related transient abdominal pain (ETAP) that have produced novel findings

Study	Year	Study objective	Study design and subjects	Key findings
Herxheimer [7]	1927	Investigate the characteristics of ETAP to determine its causation	Observational cohort study 42 individuals aged 10–20 years	Common in young people Mostly subcostal Provoked by “rhythmic shaking actions” and food, but not level of exertion Proposed to be caused by loading of the “suspensory ligaments of the stomach and intestine”
Kugelmass [5]	1937	Investigate pain characteristics and influence of age, gender, posture, and body type	Observational cohort study plus 3-month intervention involving daily breathing and posture exercises 56 symptomatic children (24 boys/32 girls, age range 7–16 years) selected from a larger cohort of 500	Pain is well localized and located in subcostal region on either side of the torso No gender difference in the experience of ETAP Not common before age 10 years but common in pubescents ‘Linear’ (ectomorph) body type more prone to ETAP as well as children with a kypholordotic postural alignment Subjects reported to have forced vital capacity below population norms 3-month intervention with breathing and posture exercises decreased symptoms of ETAP in most subjects
Capps [3]	1941	Investigate pain characteristics	Observational cohort study 55 subjects (44 male/11 female, 15–65 years)	Pain related to exertion Provoked by post-prandial state Pain variable in location Pain relieved by bending forward or applying local pressure Pain aggravated by cold weather Proposed to be caused by hypoxia of the diaphragm
Sinclair [2]	1951	Investigate pain characteristics	Epidemiological study plus observations 123 subjects (114 males/9 females, 13–36 years)	Pain located mostly upper and mid abdomen Related to shoulder tip pain Pain relieved by bending forward, deep breathing and body inversion Pain provoked by post-prandial state Pain common in activities that involve “repeated jolting” of the torso Proposed to be caused by “tugging of the peritoneal ligaments”
Plunkett and Hopkins [21]	1999	Investigate influence of four fluids on ETAP and test strategies for gaining pain relief	Cross-over trial randomized using a Latin square design 10 males (21 ± 2 years) performed a total of five sessions involving different fluids: no fluid, water, Exceed sports drink, decarbonated Coca-Cola, and a hypertonic solution of the nonabsorbable sugar lactulose During each session the subjects performed five 5-min bouts of running separated by 10 min	All fluids increased the experience of ETAP during the first three 5-min running bouts but only the hypertonic Coca-Cola and lactulose caused worsening symptoms thereafter Pain-relieving techniques: bending forward, tightening belt around abdomen, breathing through pursed lips Relaxing abdominal muscles or increasing the impact of foot strike had no effect on pain
Morton and Callister [1]	2000	Investigate the characteristics of ETAP within the past year in participants in different sports	Epidemiological study Subjects: 965 regular sporting participants (521 males/444 females, 28.5 ± 12.4 years) from six sports: running, swimming, cycling, aerobics group fitness class, basketball, and horse riding	Pain most common in activities that involved repetitive torso movement, either vertical translation or longitudinal rotation ETAP appears to be a single condition common in its manifestation to sufferers Pain is well localized and mostly occurs in the subcostal lumbar regions of the abdomen but can occur throughout the abdomen Sensation of the pain related to its severity ETAP related to shoulder tip pain Proposed ETAP is caused by a localized cramp or irritation of the parietal peritoneum
Morton and Callister [11]	2002	Investigate factors related to the experience of ETAP within the past year	Epidemiological study Subjects: same as Morton and Callister [1]	Prevalence and severity of ETAP decreases with increasing age ETAP not related to sex or body mass index Training status reduced the frequency of occurrence of ETAP but not its severity
Morton and Aune [51]	2004	Investigate the role of the thoracic spine in the experience of ETAP	Observational study involving 18 runners	Palpation of the thoracic spine (T8–12) reproduced symptoms of ETAP in symptomatic individuals
Morton et al. [20]	2004	Investigate the influence of three fluids on the experience of ETAP and its relation to abdominal ‘bloatedness’ Investigate the reproducibility of ETAP	Cross-over trial randomized using a Latin square design 40 active subjects (30 males/10 females, mean age = 21.0 ± 2.7 years) performed four treadmill running trials: no fluid, flavoured water, sports drink, fruit juice	Fruit juice (11 % carbohydrate and hypertonic) was more provocative of ETAP and feelings of bloatedness than the other fluids. No difference between the water and sports drink The fruit juice provoked ETAP independently of its bloating effect Reliability testing suggested the subjects learnt to tolerate the fluids better with practice
Morton et al. [12]	2005	Investigate the prevalence of ETAP in a single event as well as characteristics of the pain and provoking factors	Epidemiological study plus observations 848 participants in a community fun run (507 males/341 females, 627 runners/221 walkers)	27 % of respondents reported experiencing ETAP in event. 42 % of these claimed that it affected their performance in the event ETAP 3.5 times more common among runners than walkers Pain characteristics remarkably similar to those reported by Morton and Callister [1] Right side pain twice as common as left side pain Reports of ETAP decreased with age but were unrelated to sex, body mass index or time taken to complete the event Runners who consumed a large pre-event meal 1–2 h before the event were more likely to experience ETAP ETAP unrelated to the nutritional profile of the pre-event meal ETAP related to shoulder tip pain
Morton and Callister [16]	2006	Investigate the influence of ETAP on lung function	Pre-test post-test cohort study with comparison group 28 active individuals total (20 males/8 females, mean age = 23.3 ± 5.9 years): 14 in ETAP group and 14 in comparison group	Lung function was not compromised during an episode of ETAP Concluded that the diaphragm is not implicated in the cause of ETAP
Morton and Callister [22]	2008	Investigate whether localized electromyograhic (EMG) activity is increased during an episode of ETAP	Pre-test post-test cohort study with comparison group Same as Morton and Callister [16]	Localized EMG activity was not elevated during an episode of ETAP Concluded ETAP is not a muscular cramp
Morton and Callister [25]	2010	Investigate the influence of posture and body type (somatotype) on ETAP	Observational cohort study 159 active subjects (104 males/55 females, mean age = 18.6 ± 5.0 years)	Individuals with kyphosis were more susceptible to ETAP ETAP unrelated to somatotype
Mole et al. [82]	2014	Investigate the relationship between transversus abdominis function and ETAP	Observational cohort study 50 runners (28 males/22 females, mean age = 25.8 ± 7.0 years)	Participants with stronger trunk muscles and larger resting transversus abdominis size experienced less ETAP

The eligibility criteria for inclusion in the table were: the study focused specifically on ETAP, produced novel findings, and was not a case report

## Prevalence and Incidence of Exercise-Related Transient Abdominal Pain (ETAP)

Morton and Callister [1] surveyed 965 participants from six sports and reported 61 % had experienced ETAP in the past year. The prevalence of ETAP in these sports was as follows: swimming (75 %, *N* = 103), running (69 %, *N* = 439), horse riding (62 %, *N* = 100), aerobic group fitness (52 %, *N* = 126), basketball (47 %, *N* = 121) and cycling (32 %, *N* = 76). In a study of 110 triathletes, Sullivan [10] reported a similar prevalence of ETAP for running (68 %), but lower rates for swimming (15 %) and cycling (8 %). The disparity in the reported prevalence of ETAP in swimming might be explained by the swimmers surveyed by Morton and Callister [1] being very young, which, as detailed below, increases susceptibility to the pain. When age and other personal characteristics known to affect ETAP were controlled for, Morton and Callister [11] found running and horse riding to be most provocative of the pain, and cycling the least. ETAP was 10.5 and 9 times more common in running and horse riding than cycling, respectively [11].

The incidence of ETAP during a single running event has been reported by Morton et al. [12] (*N* = 848) and ter Steege and Kolkman [13] (*N* = 1,254) as 27 and 17 %, respectively. While Morton et al. [12] questioned participants immediately after completing the event, ter Steege and Kolkman [13] administered an online questionnaire within 48 hours following the event, to which some participants did not respond to for several weeks. Consequently, inaccuracies in recall may have misrepresented the incidence reported by ter Steege and Kolkman [14]. A further explanation for the differing incidences of ETAP reported in the two studies may be that ter Steege et al. [14] asked only about ETAP of ‘moderate’ or greater severity. Therefore, the subjects in this study may not have reported less intense experiences of ETAP. Smaller studies have reported the incidence of ETAP to be 21 % for a 10-km event [15] and 19 % for a 67-km ultramarathon [4]. Hence, the body of evidence suggests that approximately one in five participants in a running event may be expected to experience ETAP.

## Pain Characteristics of ETAP

While conflicting reports of the pain characteristics of ETAP appeared in early studies [2, 3, 5], more recent larger studies have demonstrated that the manifestation of the pain is remarkably similar between individuals [1, 12] and in different sporting activities [1, 11]. This is an important qualification as it indicates that ETAP is mostly a single condition, rather than a cluster of ailments [1]. Further, this suggests a single etiology, as discussed below.

### Pain Sensation and Severity

Morton and Callister [1] studied almost 600 cases of ETAP and observed that the sensation of the pain was significantly related to its severity. The pain was described as sharp or stabbing when severe, and cramping, aching, or pulling when less intense. Progression to sharp and stabbing pain with increasing pain severity has been observed subsequently in other epidemiological studies [12], clinical trials [16], and case reports [17].

When Morton and Callister questioned individuals from six sports about their experience of ETAP in the past year, symptomatic individuals reported the severity as 5.6 ± 0.2 out of 10 at its worst, resulting in 76 % having to reduce their exercise intensity when the pain was present and 12 % to stop exercise altogether [1]. In a study that targeted a single running event, sufferers rated the pain as 3.6 ± 0.1 out of 10, forcing 36 % to slow down and 6 % to stop entirely [12]. Although the pain is mostly benign, it can clearly be detrimental to performance and may compromise sports participation for some individuals [1, 12].

### Pain Location

**Fig. 1:**
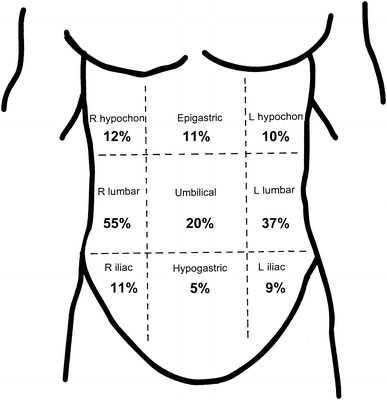
The location of exercise-related transient abdominal pain (ETAP) reported by the combined symptomatic subjects (*N* = 818) in the studies by Morton and Callister [1] and Morton et al. [12]. Note, some respondents reported ETAP in more than one position. *L* left, *R* right

In approximately 80 % of cases, the pain is described as localized rather than vague and diffuse [1, 12]. While the pain is mostly localized during an episode, it may occur in any region of the abdomen [1, 12], with pain in the mid- to upper-abdomen, especially along the costal border, being the most common site as shown in Fig. 1 [1–3, 12, 18, 19]. Right side pain has been reported as up to twice as common as left side pain [12, 16], although left side pain may be more prevalent among the young [1, 5, 11].

### Intra-Individual Frequency and Pain Reproducibility

Approximately half of the 600 symptomatic individuals surveyed by Morton and Callister [1] claimed ETAP occurred in less than 10 % of exercise sessions, and 82 % indicated it occurred no more than 20 % of the time.

The reproducibility of ETAP has been explored in two laboratory-based studies [20, 21]. Plunkett and Hopkins [21] reported poor reproducibility of the condition, although only ten subjects were involved in the study. Morton et al. [20] exercised 23 symptomatic individuals on a treadmill on two different days after consuming large volumes of fluid and found 64 % experienced ETAP in both trials, 23 % experienced ETAP in one trial, and only 13 % did not experience ETAP on either occasion. The intra-class correlation between the two trials was significant for the mean pain severity (*r* = 0.66, *p* < 0.01) but not peak severity (*r* = 0.41). The authors suggested that the between-days reliability of the pain was acceptable for repeated measure design studies, but they acknowledged that the subjective, transient, and partially unpredictable nature of the phenomenon makes studying the condition challenging [16, 20–22].

### Shoulder Tip Pain

As far back as 1951, Sinclair [2] reported an association between ETAP and shoulder tip pain (STP). In his observations on 123 athletes, 28 (23 %) reported STP in association with ETAP and in all but one case the STP was on the same side of the torso as ETAP. The significance of this observation is that the shoulder tip region—extending from the lateral third of the trapezius border to the acromion process—is the referred site for pain arising from tissue innervated by the phrenic nerve [23], which includes the diaphragm and neighboring structures. The association between STP and ETAP is informative regarding the etiology of ETAP, as discussed below.

While difficult to delineate as referred pain, Morton and Callister [1] questioned individuals about ‘non-injury-related’ shoulder pain in their study of ETAP in six sports. Fourteen percent reported experiencing sharp, well localized pain in the shoulder tip region characteristic of referred STP [23], and those who were susceptible to ETAP were significantly (*p* < 0.01) more likely to report STP. Further, STP was most common in the same sports as ETAP, albeit approximately 4–5 times less common than ETAP [11]. STP was only reported by 5 % of the 848 participants questioned by Morton et al. [12] at a running event, as compared with 27 % for ETAP, but the two conditions were significantly related. In both studies, STP was rated as more severe than ETAP but was not related to ETAP in a particular region of the torso.

## Factors Influencing the Experience of ETAP

### Personal Characteristics

In an article that appeared in the *British Medical Journal* in 1945 [24], the author asserted that some individuals are more susceptible to ETAP than others, which could be attributed to anatomical, physiological, or mechanical factors. Subsequently, investigations of a variety of personal characteristics on the experience of ETAP have been conducted [1, 5, 12, 25].

Several studies have demonstrated that the young are most susceptible to ETAP [1, 2, 26]. Morton and Callister [11] reported 77 % of active individuals under the age of 20 years experienced the pain compared with only 40 % of individuals over the age of 40 years. Both the prevalence and severity of ETAP have been shown to significantly decrease with increasing age [11, 12]. Confounding the observation that ETAP decreases with increasing age might be a reduction in activity levels, however these studies targeted an active population. While the young appear more susceptible to the pain, Kugelmass [5] reported it to be relatively uncommon before the age of 10 years.

Conflicting reports exist regarding the influence of sex on ETAP. ETAP was reported four times more frequently by females in a study by ter Steege et al. [14] of gastrointestinal complaints during long distance running. Other studies of gastrointestinal symptoms during running have also reported a higher prevalence of ETAP among females [4, 27]. Rehrer et al. [4] reported 41 % of females experienced ETAP during an ultra-marathon event compared with only 17 % of males, although the difference was not statistically significant as only 12 females participated in the event. Conversely, two studies by Morton and colleagues [1, 12] found no gender differences in the experience of ETAP when other potentially confounding factors such as age were controlled for. As the studies by Morton and colleagues [11, 12] involved a comparably larger number of cases, the weight of the current evidence suggests that there is little or no effect of gender on ETAP.

A high level of physical conditioning may decrease the experience of ETAP [2, 3]; however, the ailment is not unknown among well trained and even elite athletes [10, 12, 15]. Morton and Callister [1] observed well conditioned individuals to be as likely to report experiencing ETAP within the past year as less conditioned individuals and also to report pain of a similar severity, although better conditioned individuals reported experiencing the pain less frequently. Hence, training status might have some prophylactic benefits but in some athletes the possibility of experiencing ETAP cannot be eliminated [3].

The influence of body type and posture on ETAP has been investigated in several studies [1, 5, 11, 12, 25]. Body mass index was not related to ETAP in either of the large epidemiological studies conducted by Morton and colleagues [1, 11, 12]. Recognizing the limitations of body mass index as a surrogate for body type, Morton and Callister [25] investigated the relationship between somatotype and the experience of ETAP in 159 active young people (mean age = 18.6 years). ETAP was unrelated to somatotype, which conflicted with an early report by Kugelmass [5], who observed children with an ectomorphic disposition to be most afflicted by the pain. However, the two studies [5, 25] did concur that individuals with poor postural alignment were more predisposed to the pain. Morton and Callister [25] found individuals with kyphosis were more susceptible to ETAP (*p* < 0.01) and the extent of kyphosis and lordosis influenced the pain severity (*p* < 0.05). The implications of these observations are discussed below when considering the etiology of the pain.

### Mode of Exercise

As described previously, the experience of ETAP varies for different sporting activities, with running consistently being reported as most provocative of the condition [1, 2, 6, 10–12, 26, 28]. It has been concluded that ETAP is most prevalent in activities that involve repetitive torso movement, involving either vertical translation or longitudinal rotation, especially when the torso is in an extended posture [1, 29]. These conditions are all met in running, which explains the high prevalence of ETAP in this activity. Swimming extends the torso and involves repetitive rotation with the torso extended, accounting for a modest prevalence of ETAP. Cycling, in which the prevalence of ETAP is comparatively low, flexes the torso and involves relatively little movement.

Exercise intensity has been suggested to influence ETAP [2, 15, 28] although the pain can clearly occur during activities of low intensity such as horse riding [1]. Morton et al. [12] reported 31 % of runners experienced the pain during a walk/run event compared with only 16 % of walkers. Controlling for factors such as the age of the participants, the runners were 3.5 times more likely to report the pain than the walkers. As running also differs from walking in its movement characteristics, this would confound the potential effect of exercise intensity. Among the runners in this study, the occurrence or severity of ETAP was unrelated to the time to complete the event, suggesting exercise intensity was not important [12].

### Pre-Exercise Ingestion

Consuming food or drink before exercise has consistently been reported to evoke ETAP [1, 2, 10–12]. Consequentially, laboratory-based studies that have endeavoured to take measurements of the pain have required subjects to eat and/or drink before exercise to provoke its manifestation [2, 6, 16, 20, 22].

Fifty-two percent of respondents surveyed by Morton and Callister [1] perceived eating before exercise to provoke ETAP, and 38 % claimed that drinking before exercise provoked the pain. Sinclair [2] reported that the postprandial state could induce ETAP in 30 out of 35 cases compared with only 19 cases when prior feeding did not occur. Approximately half of the individuals who reported developing ETAP during the run/walk event studied by Morton et al. [12] claimed the pain developed shortly after ingesting fluid at a drink station. Further, an analysis of the pre-event meal of the 848 participants in this event showed a positive relationship between the volume of food and drink consumed relative to body weight and the experience of ETAP. Interestingly, ETAP was unrelated to the nutritional content of the pre-event meal with regards to carbohydrate (sugar and starch), fat, protein, water, or total energy. However, a limitation of this study was that participants were not asked whether they periodically experienced the pain and so analyses could not be performed on the symptomatic individuals only.

Two studies have shown that the composition of ingested fluid influences the experience of ETAP, with hypertonic fluids being most provocative [20, 21]. Plunkett and Hopkins [21] compared the consumption of large volumes of water, an isotonic solution, and a hypertonic solution on the experience of ETAP during treadmill running. They found that the isotonic solution evoked fewer symptoms of ETAP than water (*p* < 0.05) but the hypertonic trial was more provocative than both (*p* < 0.01). Similarly, Morton et al. [20] found a hypertonic beverage to be significantly more provocative of ETAP than isotonic and hypotonic beverages, with 83 % of subjects developing the pain after consuming the hypertonic solution compared with 70 % in the other two trials. Other studies have suggested a connection between the carbohydrate content of ingested fluid and the experience of ETAP [4, 28].

While fluid consumption appears to provoke ETAP, fluids may be better tolerated with practice. Reliability testing conducted by Morton et al. [20], which involved subjects exercising on several occasions after consuming large volumes of fluid, found that the experience of ETAP decreased in the later trials. Just as the gut may be trainable with regards to digesting and absorbing fluids during exercise [30], this may suggest that sufferers of ETAP might benefit from practicing fluid consumption during exercise.

### Inadequate Warm-up and Cold Ambient Conditions

Inadequate warm-up prior to exercise and cold ambient conditions have been suggested to provoke ETAP [3, 12, 24, 31]; however, these claims are based on anecdotal observations. For example, Capps [3] claimed that 40 out of 60 competitors in a cross-country running event that occurred in an ambient temperature of −9 °C were forced out of the event due to ETAP. Morton et al. [12] reported that equal numbers of competitors developed ETAP during the first, middle, and final third of a 14-km running event, suggesting that inadequate warm-up was not an important contributor. At this time, the influence of these factors is inconclusive and requires further clarification.

## Etiology of ETAP

Numerous theories have been proposed to explain the mechanism responsible for ETAP, however, many are based on case studies or anecdotal reports. The recent spate of investigations over the past 15 years have provided a better understanding of the characteristics of ETAP and factors relating to its provocation, allowing an evidence-based approach for appraising previously proposed theories and generating alternative explanations [1, 11, 12, 16, 20–22, 25]. Interestingly, long-held theories that have now been convincingly discredited still commonly appear in the literature [30, 32, 33]. In this section, the most prominent theories of ETAP are presented and appraised. Importantly, the consistent characteristics of ETAP reported in widely differing individuals and sporting activities suggests a single etiology [1, 32].

### Diaphragmatic Ischemia

In 1941, Capps [3] proposed that ETAP was caused by ischemia of the diaphragm. In support of this theory are sensations associated with innervation of the diaphragm. The diaphragm is mostly innervated by the phrenic nerve, which refers pain to the shoulder tip region, but the peripheral portions of the diaphragm are supplied by the lower six intercostal nerves, which could account for sharp and well localized pain in the subcostal region [34, 35].

The main evidence against this theory is that ETAP can be induced by activities of low respiratory demand, such as horse, camel, and motorbike riding, where ischemia of the diaphragm is improbable [1, 2, 36]. Further, this theory does not account for ETAP in regions of the abdomen other than the subcostal border. Finally, it has been questioned whether in non-diseased individuals the diaphragm muscle would become ischemic and fatigue before the muscles of the limbs, as this would imply central fatigue precedes peripheral fatigue [37].

Interestingly, in a study that predated Capps’ theory, diaphragmatic movements were monitored during an episode of ETAP using a fluoroscopic technique and were found to be full and unrestricted [6]. More recently, Morton and Callister [16] required subjects to perform a flow-volume loop while experiencing ETAP and found no compromise in any spirometry measures. They had hypothesized that lung function would be impaired, especially inhalation, if the diaphragm was ischemic [16]. In conclusion, diaphragmatic ischemia is an unlikely etiology of ETAP.

### Mechanical Stress on the Visceral Ligaments

Historically, the most widely accepted theory on the causation of ETAP has centred on mechanical stress being placed upon the visceral ligaments that support the abdominal viscera, especially the liver and stomach, via attachments to the diaphragm. The theory was first proposed at least as far back as the 1920s [3, 7], but was popularized by Sinclair [2] in 1951.

This theory explains several features of ETAP, most notably the high prevalence of ETAP in activities that are ‘jolting’ in nature yet of low respiratory demand such as horse riding [1, 2, 36]. Further, consuming food and fluid prior to exercise could provoke ETAP by the increased gastric mass loading the visceral ligaments [2]. Plunkett and Hopkins [21] argued that increased gastric mass would also result when consuming hypertonic beverages [20, 21, 28] due to these fluids slowing gastric emptying and thereby maintaining a larger gastric mass. As the visceral ligaments attach to the diaphragm, the theory could also explain the experience of STP. Less well established observations that lend credibility to the visceral ligament theory include the alleged therapeutic effect of body inversion or wearing a supportive belt around the abdomen [2, 21].

While the visceral ligament theory accounts for many of the characteristics of ETAP, it has been argued that there are several aspects of the pain that the theory cannot explain [38]. The observation of ETAP low in the abdomen is not consistent with the theory. Further, it is unclear why ETAP would be prevalent in swimming as this activity is not ‘jolting’ in nature and occurs in a prone position [1, 3]. It would be expected that increasing adipose stores within the greater and lesser omentum would contribute to an increased susceptibility to ETAP, as these structures attach via mesentery directly to the stomach [34], yet ETAP appears unrelated to body mass index [11, 12] or endomorphy [25]. Finally, as the ligaments are extensions of the abdominal viscera, pain arising from them would likely be visceral in nature, which is typically dull, medial and poorly localized [39], in contrast to ETAP, which is mostly sharp, lateral and well localized [1, 12].

In conclusion, the visceral ligament theory explains several features of ETAP such as its high prevalence in ‘jolting’-type activities and its relation to the post-prandial state. However, the theory has shortcomings relating to the documented pain distribution and characteristics, as well as the observation that ETAP can occur in non-‘jolting’ activities.

### Gastrointestinal Disturbances

ETAP has been commonly referred to as a gastrointestinal disturbance [4, 10, 13, 15, 27, 28, 40–42] and is commonly the most prevalent symptom reported in studies of gastrointestinal complaints during exercise [4, 15]. Despite being labeled a gastrointestinal disturbance, the etiology of the pain from a gastrointestinal perspective is poorly defined [15, 41, 43].

The few reports that have offered a gastrointestinal explanation for ETAP have focused on the pain originating from gut ischemia [13, 15, 36, 44] or distension [36]. Supportive of an ischemic explanation for the pain, splanchnic blood flow decreases by up to 80 % during exercise [13, 41]. Indeed, gut ischemia has been observed in otherwise healthy subjects, but only during maximal exercise [45], unlike ETAP which can occur during lower intensity activities.

The main reason ETAP has been labeled a gastrointestinal disturbance is due to its association with the post-prandial state [1, 2, 10, 12, 21], yet the pain is commonly experienced when no food or drink is consumed for several hours before exercise [20, 21]. More importantly, the established characteristics of ETAP are dissimilar to gastrointestinal pain, which is typically described as medial, diffuse and colicky [6, 39]. Silen [39] observed that people with gastrointestinal discomfort tend to writhe the torso to obtain pain relief, which is unlike ETAP that is relieved by reducing movement [1, 2, 12, 21, 22].

In conclusion, while ETAP is commonly considered a gastrointestinal complaint, elements of the pain are not consistent with a gastrointestinal origin.

### Muscular Cramp

In the two large epidemiological studies by Morton and colleagues [1, 12] (see Table 1), approximately one in four sufferers of ETAP described the sensation of the pain as ‘cramping’. They suggested that a muscular cramp could explain several of the features of ETAP, such as the pain occurring throughout the abdomen, and suggested the theory warranted further investigation. Subsequently, Morton and Callister [22] measured localized electromyographic (EMG) activity while ETAP was present, as muscular cramp is associated with high levels of EMG activity [46, 47]. EMG activity was not elevated at the site of ETAP during an episode of the pain, which convincingly discredited the muscular cramp theory.

### Median Arcuate Ligament Syndrome

ETAP has been referred to in the literature as median arcuate ligament syndrome [48, 49]. The median arcuate ligament is a fibrous arch that unites the crura of the diaphragm on either side of the aortic hiatus. In some people, the ligament inserts low and crosses the proximal portion of the celiac axis, which can cause compression and ischemic-related epigastric pain, especially post-prandially and during expiration [50]. While the condition does not explain several of the characteristic of ETAP, such as its manifestation in other regions of the abdomen, the anatomical abnormality leading to the syndrome is surprisingly common, being present in approximately 16 % of individuals and 30 % of young people [48].

### Neurogenic Pain

The experience of ETAP appears to be affected by poor posture, especially in the thoracic region [5, 25]. Kugelmass [5] was the first to make this observation and he suggested that poor posture might affect ETAP by altering the mechanics of the abdominal structures responsible for the pain. However, Morton and Aune [51] noted that palpating specific vertebrae in the T8–12 region, which innervates the abdominal wall, could reproduce symptoms of ETAP. ETAP could be exactly reproduced in 8 of 17 subjects assessed, and the site at which the subjects reported ETAP corresponded to the dermatome arising from the nerve root being palpated. Hence, they suggested that a neurogenic origin of ETAP should be considered.

Other reports of ETAP-like symptoms in the literature have implicated the nervous system. For example, slipping rib syndrome, in which hypermobility of the eighth, ninth and/or tenth rib results in trauma to the adjacent intercostal nerve, is a documented but often undiagnosed source of upper abdominal pain similar in nature to ETAP [52–58]. Similarly, abdominal wall nerve entrapment, in which the anterior cutaneous branch of an intercostal nerve is compressed at the site at which it reflexes sharply and pierces the abdominal musculature, can produce pain with similar features to ETAP [54, 59–62]. Finally, spinal tumors and facet joint cysts have been known to produce similar symptoms to ETAP as a result of compressive forces being placed on the intercostal nerves [63, 64].

While a neurogenic explanation does not account for all features of ETAP, such as its relation to the post-prandial state, it is notable that the intercostal nerves can be rendered vulnerable to compression as a result of a reduction in intervertebral disk height that can occur during repetitive, dynamic torso movements such as running [65, 66].

### Irritation of the Parietal Peritoneum

After studying approximately 600 sufferers of ETAP, Morton and Callister [1] suggested that ETAP might be caused by irritation of the parietal peritoneum, which is the outer layer of the peritoneum that adheres to the abdominal wall and underside of the diaphragm. They presented the following evidence for ETAP arising from this tissue: aggravation of the portion of the parietal peritoneum that adheres to the abdominal wall causes sharp, well localized pain similar in nature to ETAP [23, 67]; the portion of the parietal peritoneum that underlays the diaphragm is innervated by the phrenic nerve and gives rise to STP when aggravated [23, 68–70]; pain arising from the parietal peritoneum is accentuated by movement [39]; the parietal peritoneum traverses the entire abdominal wall which could account for the widespread distribution of ETAP [34, 71]; the parietal peritoneum is most firmly adhered to the abdominal wall along the linear alba and the greatest potential for movement is therefore in the lateral aspects of the abdomen [34]; the tension in the parietal peritoneum is increased with the torso in an extended posture; children have a proportionally larger peritoneal surface area than that of adults which might explain the high prevalence of ETAP in the young [72]; and pain arising from the parietal peritoneum relieves quickly on removal of the stimulus [23] which is similar to that observed for ETAP when activity is ceased [16].

In addition to these observations, several case reports describing ETAP have implicated the parietal peritoneum [73–75]. Dimeo et al. [73] reported the case of a 29-year-old national class distance runner with severe, recurrent ETAP in the upper right abdominal quadrant during exertion. Laparoscopy showed congenital supernumerary ligaments binding the gallbladder to the abdominal wall. Symptoms of ETAP resolved after cholecystectomy and resection of the adhesions. Similarly, Lauder and Moses [74] reported adhesions between the ascending colon and anterior abdominal wall to be the cause of recurrent ETAP in a 28-year-old triathlete. The pain resolved following surgical intervention. Several authors have also suggested that ETAP is caused by a ‘cecal slap syndrome’ in which the caecum slaps against the anterior abdominal wall during repetitive jolting actions such as running [19, 76].

Morton and Callister [1] suggested that ETAP might result from increased friction between the parietal peritoneum attached to the abdominal wall and the visceral peritoneum that overlays the abdominal viscera. They argued that friction might be increased by the visceral and parietal layers of the peritoneum being forced more firmly together by distension of the stomach, as occurs in the postprandial state, or other abdominal organs such as the liver or large intestine. Further, they suggested that exercise-mediated changes in the quantity or viscosity of the lubricating serous fluid contained within the peritoneal cavity might increase friction. The serous fluid within the peritoneal cavity is in a constant state of flux [71, 77], being derived from splanchnic blood flow, which decreases during exercise [13, 41], and draining into the lymphatic system, which is facilitated by movement of the diaphragm [78–80]. Interestingly, the fluid in the peritoneal cavity is highly responsive to osmotic gradients between it and its vascular supply [71, 77, 81], which might explain the provocative effect of consuming hypertonic beverages on ETAP [20, 21]. Indeed, results of the study by Morton et al. [20] indicated that consuming hypertonic beverages provoked ETAP for reasons other than just increasing gastric mass due to slowing of gastric emptying.

Recently, Mole et al. [82] found that individuals symptomatic to ETAP had significantly thinner transversus abdominis and poorer functional core stability than asymptomatic individuals. The authors argued that better strength and activation of the abdominal musculature, especially the transversus abdominis, might reduce abdominal content mobility leading to lesser symptoms of ETAP. This observation may lend further support to the parietal peritoneum theory, although it would also support the visceral ligament theory as better core stability might reduce loading on the visceral ligaments.

In summary, many of the characteristics of ETAP are consistent with irritation of the parietal peritoneum, and while speculative, friction on this tissue might be a plausible explanation for ETAP.

### Conclusions

The etiology of ETAP remains speculative, although progress has been made within the past 15 years regarding potential mechanisms. While some long-standing theories seem unlikely in the light of more recent findings, other novel theories have emerged. Indeed, determining the cause of ETAP is the first step towards developing procedures to manage the ailment, if at all possible.

## Management of ETAP

Many techniques and strategies have been proposed for preventing ETAP from occurring and/or gaining relief from the pain when it is present. However, as the etiology of the pain has not been well understood, many of the documented management strategies are anecdotal.

To prevent ETAP, large volumes of food and drink should be avoided at least 2 hours before exercise [12], possibly 3–4 hours [2, 21, 26, 31, 83] for those more vulnerable. During exercise, small but regular volumes of fluid might be better tolerated [32]. Hypertonic beverages should be avoided [20, 21].

Techniques that support, or restrict movement in, the torso might be helpful [21, 32]. This may be achieved by wearing a wide, supportive belt around the waist [21, 32], but improving functional core stability is more desirable [2, 6, 19, 31, 82]. Spitznagle and Sahrmann [18] reported two case studies in which exercises tailored to improve dynamic trunk stability were effective for preventing the occurrence of ETAP.

Kugelmass [5] reported that improving posture reduced symptoms of ETAP in the children he studied. Similarly, two case studies have reported symptoms of ETAP improving through a treatment regimen that aimed to improve spinal alignment and function [17, 51].

As the frequency of ETAP occurrence may decrease with improved fitness level [1, 2, 19], physical conditioning might be considered a prevention strategy. Failing these strategies, McCrory [84] comically advocated: “grow old, as stitches are less common with aging”.

To gain relief from ETAP when the pain is present, the most common techniques reported by almost 600 sufferers questioned by Morton and Callister [1] were deep breathing (40 %), pushing on the affected area (31 %), stretching the affected site (22 %) and bending over forward (18 %). Deep breathing has been reported to relieve ETAP by other authors [2, 5] although Plunkett and Hopkins [21] found that breathing shallowly but with more air in the lungs throughout the breathing cycle relieved the pain in their subjects. Pushing on the affected site might conceivably support the abdominal organs or restrict their movement and is not dissimilar to tightening a wide belt around the waist, which was reported by Plunkett and Hopkins [21] to relieve the pain. Reports of bending over forward [1, 2, 85] and stretching the affected site [1, 44] seem contradictory, which questions the efficacy of these maneuvers.

In conclusion, it is disappointing that the despite the recent research interest in the condition, innovative methods for managing the well known, potentially debilitating pain have not been determined. Indeed, the most effective strategy for relieving ETAP remains to stop exercising [5, 6, 16, 24, 32, 85], which is not always practical or desirable.

## Conclusion

ETAP is a common condition, experienced by approximately one in five participants in a running event. While the pain has been known since antiquity, it has received relatively little research interest until the past 15 years. Recent investigations have determined that the pain is especially prevalent in activities that involve repetitive torso movement, such as running and horse riding; it is typically well localized in the lateral aspects of the mid abdomen along the costal border, although it may occur in any region of the abdomen; it is related to STP; is sharp or stabbing in nature when severe, and cramping, aching, or pulling when less intense; is exacerbated by the postprandial state, especially hypertonic beverages; and is most common in the young but not necessarily related to sex, body type or training status. Greater understanding of the characteristics of ETAP has provided better clarity on the mechanism responsible for the pain; however, its etiology remains to be fully elucidated and hence strategies for managing the pain are largely anecdotal. Commonly purported prevention strategies include avoiding large volumes of food and beverages for at least 2 hours prior to exercise, especially hypertonic compounds; improving posture, especially in the thoracic region; and supporting the abdominal organs by improving core strength or wearing a supportive broad belt. Presently, irritation of the parietal peritoneum appears to best explain the ailment but further studies are needed to better understand the etiology of ETAP so as to inform the development of strategies for managing this unwelcome exercise-related pain.
